# Time is of the essence: an application of a relational event model for animal social networks

**DOI:** 10.1007/s00265-015-1883-3

**Published:** 2015-03-24

**Authors:** K. P. Patison, E. Quintane, D. L. Swain, G. Robins, P. Pattison

**Affiliations:** 1 CSIRO Livestock Industries, JM Rendel Laboratory, Ibis Avenue, North Rockhampton, QLD 4701 Australia; 2 School of Management, The University of Los Andes, Bogota, Colombia; 3 The Melbourne School of Psychological Sciences, The University of Melbourne, Melbourne, VIC 3010 Australia; 4 Present Address: Central Queensland Innovation and Research Precinct, CQUniversity, Ibis Avenue, North Rockhampton, QLD 4701 Australia

**Keywords:** Animal social networks, Temporal data, Social association, Social structure, Triad, Event probability

## Abstract

Understanding how animal social relationships are created, maintained and severed has ecological and evolutionary significance. Animal social relationships are inferred from observations of interactions between animals; the pattern of interaction over time indicates the existence (or absence) of a social relationship. Autonomous behavioural recording technologies are increasingly being used to collect continuous interaction data on animal associations. However, continuous data sequences are typically aggregated to represent a relationship as part of one (or several) pictures of the network of relations among animals, in a way that parallels human social networks. This transformation entails loss of information about interaction timing and sequence, which are particularly important to understand the formation of relationships or their disruption. Here, we describe a new statistical model, termed the *relational event model*, that enables the analysis of fine-grained animal association data as a continuous time sequence without requiring aggregation of the data. We apply the model to a unique data set of interaction between familiar and unfamiliar steers during a series of 36 experiments to investigate the process of social disruption and relationship formation. We show how the model provides key insights into animal behaviour in terms of relationship building, the integration process of unfamiliar animals and group building dynamics. The relational event model is well suited to data structures that are common to animal behavioural studies and can therefore be applied to a range of social interaction data to understand animal social dynamics.

**Electronic supplementary material:**

The online version of this article (doi:10.1007/s00265-015-1883-3) contains supplementary material, which is available to authorized users.

## Introduction

Analysing animal social structure based on social associations has become increasingly popular in the research literature (Lusseau and Newman 2004; Croft et al. 2011; Whitehead and Lusseau 2012). The approach stems from earlier animal-related research by zoologists and sociologists (Chase 1974; Hinde 1976). Quantifying animal social association underpins ecological and evolutionary knowledge related to biological processes such as mate selection (Psorakis et al. 2012), social learning (Kendal et al. 2010) and the potential for disease transmission (Hirsch et al. 2013). Recent research has adopted social network concepts to study the effect of social associations on individual and population level outcomes (Granovetter 1973; Snijders 1996; Newman and Park 2003; Burt 2004). Social network research studies the relationships between individuals and the effects these relationships have on individual outcomes as well as on the overall social system (Wasserman and Faust 1994). Individuals could refer to a sole animal, subgroup or population, and the relationships that connect them, such as a social interaction, kin relationship or shared habitat. Understanding how social relationships are created, maintained and severed is a critical avenue for research on human social networks.

Similarly, understanding the social processes that lead to the creation and disappearance of relationships between animals is critical because changes in social relationships have both ecological and evolutionary significance (Psorakis et al. 2012). Many fission-fusion societies exist in wild populations, where group composition changes depending on internal and external factors such as mating season (Psorakis et al. 2012), food resources (e.g. zebra, Sundaresan et al. 2007), or diurnal patterns (e.g. primates, van Schaik 1999). Conversely, cattle naturally exist within stable social systems where group composition rarely fluctuates (Reinhardt and Reinhardt 1981). However, livestock production systems rely on regrouping individuals according to production parameters (e.g. age, pregnancy status, weight), which can result in social disruption and instability (Zayan 1991). Implications of this disruption can result in agonistic behaviour, changes in maintenance behaviour and physiological stress (e.g. Kondo and Hurnik 1990; Hasegawa et al. 1997; Gupta et al. 2008). Thus, the process of social re-stabilisation is important for the animal’s welfare.

Human social relationships are typically considered as states (e.g. friend of, collaborates with) that are conceptually distinct from specific interactions (Robins 2015), even though a human social relationship usually entails some regularity of dyadic interaction. Animal social relations, on the other hand, are largely inferred from interactions, so that the observation of association patterns over time is evidence for the existence (or absence) of a social relationship between animals. For example, social relationships can be inferred from displays of affiliative behaviour, such as allogrooming, providing protection and maintaining close proximity (Newberry and Swanson 2001). Consequently, most data used in animal social network analysis are based on interactions between animals rather than the reports of states of social relations that are typical of many human network studies. While recent technological advances in animal behaviour monitoring are enabling researchers to obtain very fine-grained information about the temporal and spatial dynamics of animal movement and behaviour, standard statistical frameworks in network analysis require the data be aggregated into one cross-sectional dataset, or at best into panels of data at specific time points, thereby removing detailed information about timing and sequence (Blonder et al. 2012). This time sequence information, however, is critical to animal studies for understanding the processes of specific social behaviours, such as reciprocity (Trivers 1971) or hierarchy formation (Chase 1982). Analysing data with respect to timing and sequence can also provide valuable information on critical short-lived events that occur suddenly, such as a mating event, which can potentially be missed when aggregating data. Developing event-based models that allow analysis of sequential data are gaining a new popularity in the social networks literature by providing a way of handling long sequences of exchanges within the context of a relational system. Such models have come to prominence with the availability of digital data, where the data does not contain information about relationship states but comprises sequences of transactional exchanges (or ‘relational events’) within dyads.

In this paper, we describe a statistical model derived from these new social network methods. The model enables the analysis of fine-grained observational data on animal associations over time without the need for aggregation, thus avoiding the loss of temporal and sequential information. The relational event model that we introduce is based on Butts’s (2008) framework and is equivalent to a multinomial conditional logistic regression. The model predicts each association between two animals (e.g. physical proximity, grooming) based on a history of associations between these two animals; the characteristics of the animals; and previous associations between each of the animals and other animals in the network. Because we can use different lengths of history of associations to predict the existence of future associations, the model provides insights into how recent patterns of associations deviate from or reproduce patterns of association that have occurred over longer time frames.

More specifically, we present an application of the relational event model to a unique dataset of behavioural data among familiar and unfamiliar steers collected via proximity loggers during a series of 36 experiments, each lasting approximately 7.5 days. We aim to understand the social disruption generated by the introduction of an unfamiliar animal into an existing social context formed by two familiar animals. We specify statistics to capture the influence of recent history (1 h) versus more distant history (1 day) of interactions among familiar animals and with the unfamiliar animals on the emergence of relationships and groups. Our results provide evidence of disruption created by the introduction of the unfamiliar animal into the pair of familiar animals and highlights the processes through which social groups emerge and consolidate in the face of disruption.

## Approaches to modelling social networks

Statistical modelling of social networks implies accounting for the dependencies inherent in social network data that reflect important social processes, such as reciprocity or transitivity. Three statistical frameworks are currently available for the modelling of social network data in a way that accounts for the dependencies in the data: exponential random graph models (ERGMs), stochastic actor oriented models (SAOMs) and the relational event model (REMs). Each of these frameworks is best suited for different types of data structures. The ERGM framework is best suited for cross-sectional observations of social networks, usually taken at one point in time (Lusher et al. 2013). A typical data structure for an ERGM model is one directed or undirected network composed of binary relationships between actors. The SAOM framework is best suited for the modelling of a network that evolves over time, captured using panel data at discrete points in time (van de Bunt and Groenewegen 2007; Snijders et al. 2010). SAOM also requires binary ties, directed or undirected. There are longitudinal versions of ERGM’s for panel network data, but SAOMs are preferred, especially when nodal attributes also evolve across time. Hence, both ERGM and SAOM require a network composed of binary ties captured at one point in time as a cross section or at a limited number of discrete time points. Because this requirement entails aggregation of sequences of events that occur in continuous time into one or several cross sections, the timing and sequence of the events are lost when using ERGMs or SAOMs with relational event sequences. For example, the stream of events obtained through a logger would need to be aggregated into one or several cross sections, which would remove information about the timing and the sequence of events.

By contrast, the relational event model (Butts 2008) is specifically designed to model sequences of relational events that occur continuously through time. More specifically, the relational event framework enables direct modelling of sequences of relational events without aggregating them into cross sections. Relational event modelling was first proposed by Butts (2008) to describe radio communication pathways and similarly applied by Brandes et al. (2009) to analyse patterns of political conflict. More recently, there has been an active and growing interest in developing and applying relational event models for social data (de Nooy 2011; Stadtfeld and Geyer-Schulz 2011; Vu et al. 2011; DuBois et al. 2013; Lerner et al. 2013a, b; Quintane et al. 2013), including the development of an R package, ‘relevent’, by Butts (2014), a tool kit of syntax to fit relational event models. The term *event* relates to any form of social action between two or more individuals. The approach incorporates relational effects that have been used in cross-sectional and longitudinal models for interaction or relational data and, hence, have become familiar in models for network structure (e.g. Snijders et al. 2006). Such an approach has been noted in the animal science literature (e.g. Blonder et al. 2012; Psorakis et al. 2012; Pinter-Wollman et al. 2014) although to our knowledge has not been applied to animal association data.

## Model overview

The current form of the model is a conditional logistic regression model that uses an ordinal form of event history analysis (e.g. Blossfeld and Rohwer 1995) to analyse sequences of events in the past to predict the possibility of future events. Markov chain models have previously been used to analyse transition probabilities (e.g. Azzalini 1994), which consider random processes and use current event states to predict future events without considering the history of prior events. The current model differs from the Markov approach as each potential event is considered to be independent of all other events but conditional on the collection of events that have occurred in the past. In other words, the model uses the structure of past events to predict the occurrence of events in the future. Importantly, the next event is assumed dependent on the patterns of relational exchanges in the past, hence the terminology *relational events model*.

### Model description

The current model specifies the probability of a potential event between individuals *i* and *j* at time, *t*. The sequence of events up to and including the actual event at time *t* is:

$$ {A}_t=\left({a}_m:m\le t\right), $$

where *m* is the time at which the event *a* occurs and *a*_*m*_ is an encounter between individual *i* and *j* at the *m*th moment in time. The potential event at time *t* that involves an encounter between *i* and *j* is denoted by Y_*ijt*_ which has the value 1 if individual *i* encounters individual *j* at time *t* and has the value 0, otherwise.

The probability of an event occurring at time *t* based on an earlier event at time *t′ < t* is the probability of all possible events that could have occurred in the time interval from *t′* to *t* (i.e. the joint likelihood that none of the possible events occurred in the interval between *t′* and *t*) (Butts 2008). The realisation of all actual and possible events in the data set provides a complete description of all encounters (Blossfeld and Rohwer 1995).

The probability of the next event is described by:

$$ p(a)={\lambda}_a/\left({\displaystyle {\sum}_{a\prime }{\lambda}_{a\prime }}\right), $$

where λ_a_ is a rate parameter associated with event *a* and the events *a*′ run over all possible events. The parameter λ_a_ is dependent on the prior history of events and exogenous covariates, such as characteristics of the individuals, and can be parameterized in the form:

$$ {\lambda}_a= \exp \left({\displaystyle {\sum}_h{\theta}_h{s}_h}\right), $$

where the statistics *s*_h_ is determined by the prior history of events and exogenous covariates (described below) and θ_h_ are corresponding parameters. The parameters can be used to explain how prior events are related to future events: a large positive (or negative) value of θ_h_ indicates that an event *a* is more (or less) likely if the statistic *s*_h_ summarising relevant prior events is high.

### Model components

Our particular relational event model used time-stamped association data collected using proximity loggers between any set of three individuals. The loggers record the duration and frequency of all close proximity encounters, thus each recorded event has a beginning and an end, referred to as onset events and offset events, respectively. A sample of the data stream is shown in Table 1. Onset events relate specifically to the creation of a tie between two or more individuals and can characterise the nature of a social relationship. For example, a consistent pattern of onset events between the same pair may represent affiliative behaviours such as mutual grooming or grazing within close proximity. There were a number of different onset event types involved in each interaction (see below), where the event sequence of prior events was used to determine the event type. Offset events describe the dissolution of an existing event, but in order to present a simpler model as an illustration of the method, they are not modelled in this article and are the subject of ongoing work (see Discussion). Offsets, however, were still considered as a function of the onset event sequence, as every onset event has an associated offset event and the occurrence of offset events were important to determine the type of onset event that occurred.

**Table 1:** An example of the proximity logger data stream

Cow ID	Encountered cow ID	Start date	Start time	End date	End time	Duration (seconds)
1	2	9/03/2009	18:49:34	9/03/2009	18:49:35	1
1	2	9/03/2009	18:52:57	9/03/2009	18:56:00	183
1	2	9/03/2009	18:56:05	9/03/2009	18:56:21	16
1	2	9/03/2009	18:56:48	9/03/2009	18:58:38	110
1	2	9/03/2009	18:56:05	9/03/2009	18:56:21	16
1	2	9/03/2009	19:01:03	9/03/2009	19:01:06	3

### Predictor variables

**Fig. 1:**
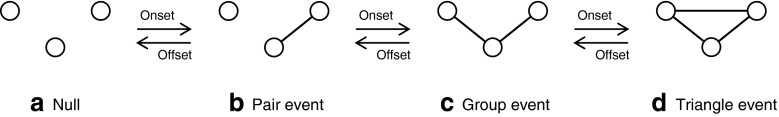
The event types that could potentially occur between three animals. The *solid lines* represent a tie between two individuals

The model included an individual attribute factor *familiarity* between individuals (whether or not the individuals knew each other prior to the start of the study). *Event type* was included in the model, based on the number of individuals involved in an event and their configuration at the time. Event type was based on triadic association where there were three possible event configurations: (i) an event involving an association of an isolated dyad (a pair event); (ii) an event involving a simultaneous onset of one animal with the other two (a group event); or (iii) an event involving a pair-wise onset among all three animals at once (a triangle event) (Fig. 1). A pair event involved a change in state from no existing contact to the creation of a single tie between two individuals, while a group event was conditional on the contemporaneous existence of a pair event. A triangle event was formed when a tie was created between the two animals that were not connected in the prior group event, thus all three individuals were in contact at the one time. A triangle event was conditional on the contemporaneous existence of a group event.

In this article, we present separate models to predict pair and group events. Given that our models are for onsets only, a model predicting the next pair event is conditional on the current situation being one of non-contact; and a model for the next group event is conditional on the current situation being one of a pair. For example, if the current situation involved two animals connected in a pair event, the next onset event could only occur between one animal already involved in the current event and the third previously unconnected animal (Fig. 1). In other words, we are predicting pair and group events for particular animals based on counts of those animals’ past associations in pair and group events. Such a model can tell us how past patterns of onsets relate to future onset events for particular animals. However, conditional on the current presence of a group, an onset necessarily implies the occurrence of a triangle with probability 1, so triangle events cannot be modelled using only onsets. In what follows, we ignore triangle-based models, leaving them for the development of more complex onset-offset models. Fortunately, as described below, triangles were quite rare in our data.

The predictors then were the frequency of prior events over a given period. Two periods were modelled simultaneously to capture both short and longer term regularities in association patterns: the immediate past hour and daily patterns. The time frames (hour and day) were chosen to reflect the behavioural patterns of the species investigated in the case study (in this application, shorter time frames did not result in different statistics or significance patterns; data not shown). Both time frames have potentially important implications for social processes. In human relational event models, different time frames have been shown to be important (Quintane et al. 2013). Short-term patterns reflect recent animal activity while daily patterns represent regularity in association patterns and long-term contact preferences. Each time interval considered prior pair events and prior group events as separate predictors in the model; the distinction between event types was important, since pair events indicate isolated dyadic activity, whereas group events relate directly to associations involving all three individuals and the potential for group formation. While there is some correlation between the two time frames, as what happens in the preceding hour is nested within what happens in the preceding day, the hourly statistics capture interaction patterns that deviate from daily patterns, thus identifying short-term activity bouts, for example biologically significant short-lived events such as a mating event, that are not representative of daily activity. This approach differs from methods that rely on aggregation, as each event is considered within the model and the outputs are summarised by meaningful time frames as opposed to presenting averages per nominal time frame.

### Model input

**Fig. 2:**
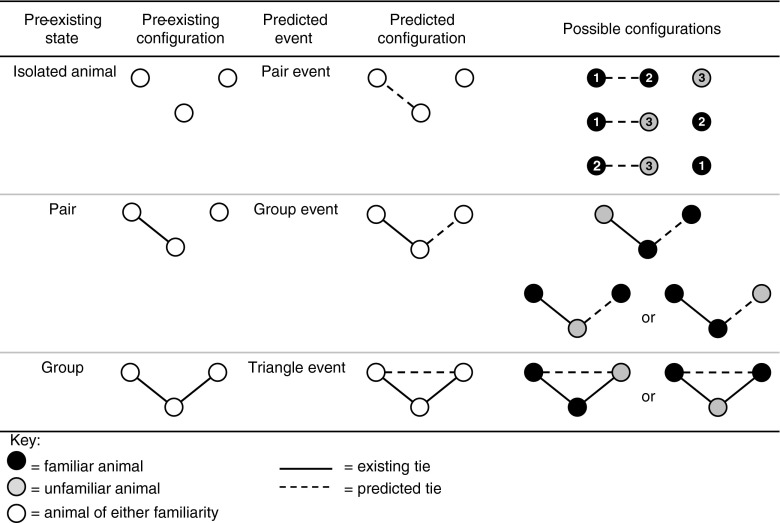
The three onset event types that could occur between three animals, their prior configuration state and the predicted configuration possibilities

Prior to transforming the association data from the proximity logger data stream into an event sequence, events that occurred at exactly the same time were temporarily distinguished from one another by adding 1 s to the second event (there were 24 events in our data set that required adjusting). A custom-developed java application was used to transform the association data into actual and potential onsets and produced statistics based on the history of prior events (to view the Java syntax, please see the supplementary online material). Potential onsets are onsets that could have occurred at the same time as the onset being modelled but did not. For example, assuming that the observed onset was an interaction between animals 1 and 2, the potential onsets were (a) an interaction between animals 2 and 3 or (b) an interaction between animals 1 and 3. Onsets were defined over the full observation period for each model (i.e. a day). The program calculated statistics for each event or potential event for (i) the familiarity of the animals involved, (ii) the type of the event that occurred (either pair, group or triangle event) and (iii) statistics based on the frequency of relevant prior events. The possible onset event configurations are shown in Fig. 2. For pair events, three events could potentially occur, a familiar pair between animals 1 and 2, an unfamiliar pair between animals 1 and 3 and an unfamiliar pair between animals 2 and 3. For a group event, there were only two possible events that could occur, a group event where the prior pair involved the unfamiliar or an event where the unfamiliar animal was involved with the creation of a tie with one of the two familiar steers in an existing dyad to form a group. Triangles formed from no prior contact did not occur in the current data set (data not shown).

The relational event model was fitted with conditional logistic regression using SPSS (to view the SPSS syntax please, see the supplementary online material). The model calculated the probability of the next event as a function of counts of previous events between the two animals over the time period. The next event in the sequence of events was considered the dependent variable. For example, when a pair event was being predicted, all previous pair events were placed in a sequence with respect to their temporal ordering and each pair in the sequence was considered a dependent variable in the model. The time period used in this application was one day, thus the data for each day started and finished on the same day, and data recorded in the previous day was not considered in the day being modelled. On the day of introduction, the statistics contained all of the events that occurred prior to the event being modelled on that day, thus the day of introduction was treated the same as all other days. As there was no daily history at the time of introduction, the model considered all the events that had occurred up until the event being modelled for the daily statistics. The statistics were normalised in line with Quintane et al. (2013), so as to be comparable in scale over the time period.

A parameter was estimated for each statistic, or effect, in the model, and each model involved five effects: (i) unfamiliarity (i.e. whether the animal was the unfamiliar animal—see below); (ii) prior pair events (past hour); (iii) prior pair events (past 24 h); (iv) prior group events (past hour); (v) prior group events (past 24 h). Two separate models were fitted on a daily basis to predict the probability of pair events and group events separately.

## Case study: cattle associations and social disruption

The case study formed part of a larger research programme exploring relationship development in cattle (Patison et al. 2010a, b). Social association data were recorded directly onto proximity logging devices (Sirtrack, Havelock North, New Zealand) that were mounted onto a collar and fitted around each individual’s neck. The loggers are UHF radio telemetry devices that continuously record the frequency and duration of close proximity contacts, thus providing a continuous stream of time ordered association data. Limitations were imposed on the data to control for known discrepancies in the logger data, for example, contacts less than 1 s were removed as they can erroneously occur when two loggers are at the edge of their detection zones (Prange et al. 2006). Thus, the minimum contact length was 2 s; this duration was chosen to capture short fleeting associations between unfamiliar individuals.

**Fig. 3:**
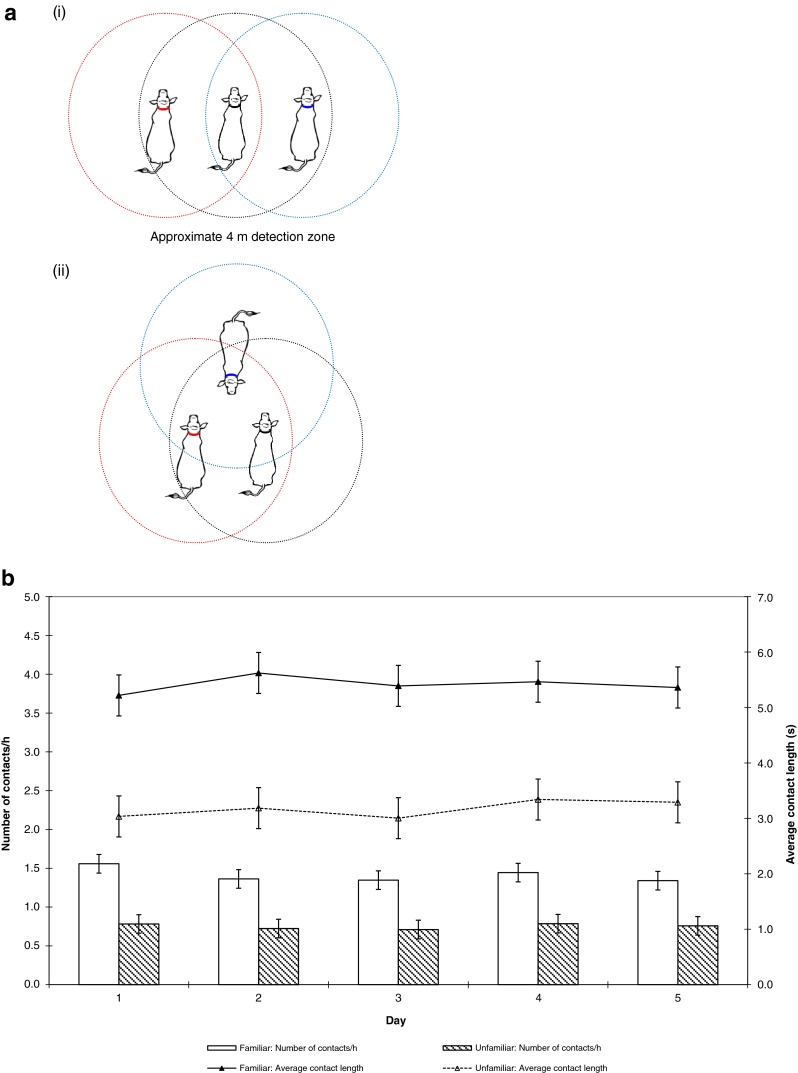
A case study applying a relational event model to relationship development in cattle using proximity loggers to record all close proximity encounters between a pair of familiar steers with a newly introduced unfamiliar steer over 5 days. Proximity loggers continuously record the date, time and duration of all close proximity encounters in sequence. A java programme was used to transform the data from each of the three loggers into a single event stream and classify each encounter based on one of three event types based on the number of individuals involved the encounter. **a** The proximity loggers recorded all encounters that occurred within a 4m detection zone; this range is equivalent to two body lengths of a collared animal. Encounters detected within this range relate to all forms of social behaviour, such as grazing and resting within close proximity (as in *i*), investigative behaviour (as in *ii*) and grooming events. **b** A summary of the proximity logger data prior to transformation with the Java programme. Aggregating the data provided a basic overview that there were more contacts (*bars*, ±SED) of longer duration (*lines*, ±SED) between familiar steers than familiar-unfamiliar contacts over the 5-day period (data are square root transformed interaction means). Being familiar strengthens group cohesion and provides essential social support, which in this case, may have contributed to the low level of association with the unfamiliar animals and the suggestion that the unfamiliar animal was being excluded from the familiar pair. The daily patterns showed no evidence that the unfamiliar was integrated into the pair; the time taken for a new individual to be accepted into a group depends on various factors, such as the species, sex, number of individuals and the space available.

Additionally, processing the data as a time sequence allowed for reciprocal issues to be rectified, where previous studies have relied on pre-processing to manipulate the data (e.g. Swain and Bishop-Hurley 2007; Hamede et al. 2009). For each collar, the transmitting output power was set to UHF 40. The detection range was set to record contacts less than 4 m to represent meaningful social encounters that occurred within two body lengths of a collared animal (see Fig. 3a). The animal’s body will absorb a portion of the signal, thus selecting a range longer than one body length ensured that contacts initiated from the rear of the animal would be detected. Encounters detected within this range relate to all forms of social behaviour, such as investigative behaviour, grazing and resting within close proximity and grooming events.

Each recorded contact was identified as an event, where spatial proximity was used to infer social association. Inference of sociality from proximity is based on the ‘gambit of the group’ theory, which states that if two individuals are within proximity, then they are interacting in some way (Whitehead 1995; Franks et al. 2010), thus temporal and spatial dynamics reflect social affinity (Coussi-Korbel and Fragaszy 1995). The resultant set of events depicts a *proximity network*, where edges are created when individuals approach each other and disappear when they move apart (Blonder et al. 2012). Thus, a priori definitions (being within 4 m for longer than 1 s) were used to define interaction thresholds.

Two groups of 2.5-year-old Brahman (*Bos Indicus*) steers were used as the animal model. Cattle were chosen as they provided an opportunity to manipulate social structure; cattle are known to exhibit strong social connections (Reinhardt and Reinhardt 1981). To investigate how existing relationships are affected by social instability and to identify association changes related to relationship formation, a group of familiar and unfamiliar animals formed the basis of the study. The first group comprised 24 animals that had been together for 12 months prior to the study, forming the familiar group of steers. The second group comprised 42 animals that had no previous interaction with the familiar group and formed the unfamiliar steers. Over 3 deployments between February and March 2009, association data recorded by proximity loggers were collected from 36 triads, each located in a 1.5 ha plot with a minimum 20 m buffer between plots. Twelve triads per deployment were formed using a step-wise approach: pairs of familiar steers were monitored for 2.5 days before a randomly selected animal from the unfamiliar group was introduced to the pair. Unfamiliar animals were introduced into the plots at approximately the same time. The first recorded encounter between either familiar steer with the unfamiliar steer was classed as the time of introduction. Associations between the triad were monitored for a further 5 days.

The familiar steers were re-used in consecutive deployments following a minimum 7 days re-stabilisation with original group members, while the unfamiliar steers were only monitored once to ensure that each familiar pair was only grouped with steers that were completely unfamiliar. We hypothesised that familiar steers would maintain closer proximity and therefore record more contacts with themselves than with the unfamiliar animal due to their existing relationship, but these encounter differences would decrease as the triad developed a stable and familiar relationship.

From previous experiments (Patison et al. 2010a, b), all familiar steers had experience with being paired with both familiar and unfamiliar individuals, thus there were considered to be no individual-level effects due to familiarity or unfamiliarity with the experimental conditions. Additionally, the same pair of familiar steers was never used more than once to form a triad. Thus, each triad in the experiment was considered to be independent.

### Data processing

The first encounter recorded between the unfamiliar steer with either of the familiar steers was taken as the time of introduction and classified as time zero in the model, with all prior data removed. The time of each encounter was defined as the number of seconds that had elapsed since the time of introduction.

Two separate methods were used to process (i) the data used to derive the basic proximity logger statistics and (ii) the data being analysed by the relational event model. The data used to calculate the descriptive statistics were processed on a pair-wise basis to facilitate the statistical analysis. To ensure that there was no overlap recorded by loggers in a triad, the reciprocal contacts were compressed using the same method as Patison et al. (2010b) and Hamede et al. (2009). A single file of contacts were created for each pair within the triad and further refined by classifying all unfamiliar contacts into one category, regardless of which familiar animal was involved in the contact. Combining the unfamiliar contacts into one category meant there were twice as many unfamiliar contacts relative to the one familiar pair per triad and would therefore over-represent the proportion of contacts involving the unfamiliar. To overcome this, the total number of unfamiliar contacts per triad was averaged between the two unfamiliar pairs to represent the average number of unfamiliar contacts per pair within the triad.

The number of contacts per hour and the average contact length were analysed for treatment and day effects using a repeated measures analysis of variance (Rowell and Walters 1976) in Genstat 12th edition (Payne et al. 2009), with day as the within-subjects effects and familiarity as the treatment blocked by triad within deployment. Differences were considered significant at the 5 % probability level. To meet the distributional assumptions of the statistical analyses, residual and normal probability plots were inspected for normality. The large proportion of short duration contacts and infrequent contacts per hour produced positively skewed distributions. A square root transformation provided the closest fit to normality and was applied to all proximity logger data prior to analysis. All statistical results presented in the text are back-transformed values.

The data used as input into the relational event model were processed by assigning each animal in each triad a unique animal identification code, from 1 to 3, to represent the identity and familiarity status of each animal: animals 1 and 2 represented the familiar animals and animal 3 was coded as the unfamiliar animal. Events were also coded to identify which pair was involved in the encounter, with a familiar pairing between animals 1 and 2 coded as a contact event of type ‘1’ and the unfamiliar pairings between animals 1 and 3 and animals 2 and 3 coded as types ‘2’ and ‘3’, respectively.

## Results

### Proximity logger summary

Aggregating the proximity logger data showed a basic trend that there were more contacts between the familiar animals than contacts with the unfamiliar steer (Fig. 3b), and that the greatest level of interaction occurred on the day of introduction (Table 2). There was no effect (*P* > 0.05) of day on the number or length of contacts between individuals within triads.

**Table 2:** There were more contacts, regardless of familiarity, on the day of introduction than any other days, even though it comprised only half a day (means not followed by a common letter are significantly different at *P* = 0.05). It is suggested that the greatest level of investigation and social stress occurred on the day of introduction

Day	Number of contacts/hour
1	1.17 (1.37)^a^
2	1.04 (1.09)^b^
3	1.03 (1.06)^b^
4	1.12 (1.24)^c^
5	1.05 (1.10)^b^
SED	0.05

### Model results

A summary of the event data per pair is shown in Table 3, which identifies all familiar pairs recording contacts each day post-introduction. The unfamiliar pairings, however, did not always record contacts each day, thus there was some level of avoidance or lack of active association between specific pairs. The lower number of events on day 1 was due to the day of introduction comprising only half a day.

**Table 3:** The average number of onset events recorded between each pair and the proportion of triads that recorded onset events (Pair 1 represents two familiar animals; Pairs 2 and 3 represent a pairing with the unfamiliar individual)

Day	Pair	Average number of events per pair	% of triads that recorded events
1	1	25.4 (2.80)	100 %
	2	9.3 (2.40)	89 %
	3	9.8 (2.07)	86 %
2	1	48.8 (5.00)	100 %
	2	20.6 (4.76)	86 %
	3	21.5 (5.93)	94 %
3	1	48.6 (5.41)	100 %
	2	23.1 (5.97)	83 %
	3	28.5 (5.57)	83 %
4	1	54.8 (5.77)	100 %
	2	23.2 (5.14)	92 %
	3	23.8 (5.57)	83 %
5	1	47.4 (4.87)	100 %
	2	22.1 (4.26)	81 %
	3	25.8 (6.15)	92 %

A summary of the events identified by the model is shown in Table 4, quantifying the total number of pair events, group events and triangle events. Generally, the number of pair and group events was consistent across days with the greatest number of all three event types recorded on day 4. Overall, there were consistently more pair events than group events. The occurrence of triangle events was rare and constituted only a fraction of the total number of events. Even though offsets were not modelled in this application, the numbers of offset event types are shown in Table 5 to provide a summary of the number of offset events relative to onset events, as the number of offset events determines the potential for future onset events.

**Table 4:** A summary of the number of events per type identified by the relational event model for onset events

Day	Onset event types			Total
	Pair	Group	Triangle	
1	1296	170	50	1517
2	2757	298	70	3126
3	2835	285	34	3155
4	3030	455	83	3569
5	2816	309	72	3198

**Table 5:** A summary of the number of offset dissolution events identified by the relational event model

Day	Offset event types			
	No event	Pair dissolution	Group dissolution	Triangle dissolution
1	1297	1466	130	12
2	2758	3055	220	20
3	2836	3120	176	5
4	3031	3485	309	22
5	2817	3125	236	19

#### Predicting future pair events

Overall, there was a negative effect of the unfamiliar animal (Table 6), indicating that a future event between two animals was less likely to involve the unfamiliar animal. Likewise, being familiar with another animal was associated with an increased probability of a future association. The strength of this effect varied across days; there was a decreasing trend from the day of introduction until day 4, which was followed by peak on day 5.

**Table 6:** Parameter estimates and standard errors predicting future pair events

Predicting future pair events		Day									
		1		2		3		4		5	
Parameter		B	SE	B	SE	B	SE	B	SE	B	SE
Frequency of prior events											
Unfamiliarity effect		−0.49	0.09**	−0.35	0.05**	−0.43	0.05**	−0.09	0.05	−0.52	0.05**
Prior pair events:	Past hour	−0.50	0.33	2.67	0.35**	2.25	0.34**	2.32	0.33**	2.89	0.35**
	Past day	2.59	0.24**	1.96	0.12**	2.15	0.13**	2.35	0.12**	1.88	0.11**
Prior group events:	Past hour	0.43	0.20**	0.14	0.16	0.78	0.15**	0.24	0.14	0.59	0.18**
	Past day	0.62	0.21**	0.31	0.13*	0.11	0.12	0.03	0.12	0.44	0.13**

*P < 0.05; **P < 0.01

There was a strong and positive effect of prior pair events leading to future pair events between the same animals (Table 6). This effect was consistently strong across all days for both the short and long term, except for a negative but non-significant short-term effect on day 1. The more two animals had encounters in the past the more likely they were to have encounters in the future. This effect suggests that repeated encounters involving two animals reflect a relationship between them: the encounters signify both a pattern of past encounters and the expectation of future ones. The lack of a significant short-term effect on day 1 suggests that there was some disruption caused by the introduction of the unfamiliar as the same pair events were not repeated in the future. The long-term effect on day 1, however, was positive and significant and in combination with the positive and significant pair effect on subsequent days indicates that overall prior contact between a pair leads to future contact.

There was an enhanced probability of an encounter between two animals if the same animals had encountered each other in the past day as a part of a group event (Table 6), thus if two animals were involved in a group event, there was a greater probability that the same animals would be observed together as a pair in the future. This effect was variable over the 5 days, but was generally most notable on the day of introduction, when both the short and daily trends were positive. Together with the prior pair results, these effects suggest that the pattern of future contact between two animals is affected positively by the same two animals being within close proximity in the past, either as an isolated pair or as a function of a more complex encounter involving a third animal.

#### Predicting future group events

In the model predicting future group events, there was a positive and consistent effect of the unfamiliar animal over the 5-day period (Table 7). This result indicates that future group events were created by a tie involving the unfamiliar: either the pre-existing pair creating the foundation of the group event involved the unfamiliar or the unfamiliar approached a familiar animal while it was within close proximity to the other familiar animal. This effect is confirmed by the summary results shown in Table 8, where the majority (between 70–89 %) of group events were formed from the existence of a pair event between the two familiar animals.

**Table 7:** Parameter estimates and standard errors predicting future group events

Predicting future pair events		Day									
		1		2		3		4		5	
Parameter		B	SE	B	SE	B	SE	B	SE	B	SE
Frequency of prior events											
Unfamiliarity effect		0.98	0.26**	0.29	0.17	0.88	0.20**	0.36	0.14*	0.44	0.17*
Prior pair events:	Past hour	−2.07	1.30	−1.60	1.26	−0.69	1.53	0.29	1.20	0.76	1.55
	Past day	2.35	1.06*	1.30	0.51*	1.80	0.78*	0.39	0.47	1.40	0.54**
Prior group events:	Past hour	1.19	0.56*	−0.08	0.37	−1.00	0.40*	0.84	0.38*	0.61	0.38
	Past day	−1.57	0.75*	1.20	0.42**	2.27	0.55**	1.49	0.45**	1.08	0.47*

*P < 0.05; **P < 0.01

**Table 8:** The number of group events formed from pre-existing familiar pairings and pre-existing unfamiliar pairings per day

Day	Existing dyad		Total
	Familiar	Unfamiliar	
1	125	45	170
2	265	33	298
3	222	63	285
4	317	138	455
5	234	75	309

The frequency of pair events in the past day leading to future group events had a strong and positive effect (Table 7). In other words, the more two animals interacted, the more likely were group events involving proximity between those two animals on that day. This effect was strongest on the day of introduction yet was variable over the remaining 4 days, suggesting that there was a high level of association on day 1, which settled out from day 2 onwards.

**Fig. 4:**
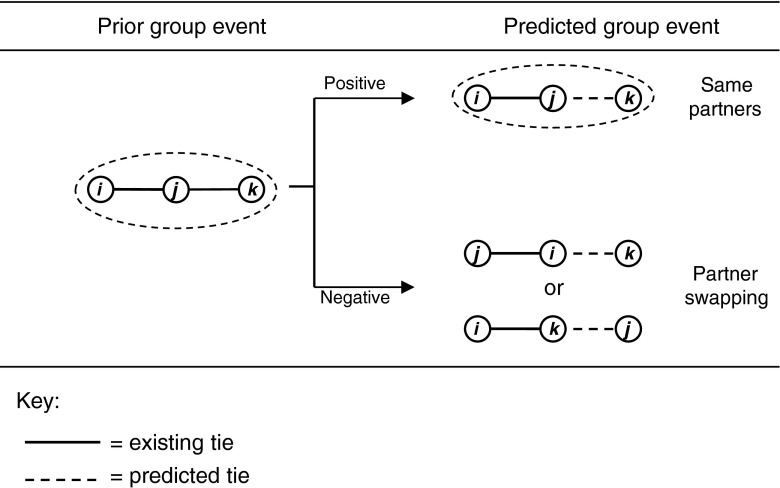
A description of the positive and negative significant parameter effects when the onset of a group event was predicted from the sequence of prior group events. A negative parameter indicates that the predicted configuration was different from the previous sequence and represents a partner swapping event

The propensity for group events to lead to future group events was variable (Table 7). On the day of introduction, the short-term effects were positive yet the daily effects were negative. The positive short-term effects indicate that the group event was created by the addition of the third animal to the same pair of animals that were tied together in past group events, which suggests short-term group building behaviour. While the negative daily effect suggests that the pair configuration of future group events was different to that of the past, or partner swapping (see Fig. 4). Taken together, the results for day 1 suggest instability within the triad resulting from the disruption caused by the introduction of the unfamiliar steer.

From day 2 onwards, the pattern of prior group events leading to future group events was also variable between short-term and daily patterns. The daily effect was consistently strong and positive. Indeed, being within close proximity in the past means it is more likely that the same pairs will be in close proximity in the future: this effect suggests stability. The change from a negative effect on day 1 to a positive effect on consecutive days suggests that the same pairs were remaining together in future group events, which could suggest relationship building between the unfamiliar steer with one of the two steers. The hourly effects, however, were not consistently repeated and alternated between negative and positive effects on days 3 and 4, respectively. These variable patterns are difficult to explain, but may indicate a continuing instability in the triad as the unfamiliar tried to be incorporated into the familiar pair, perhaps unsuccessfully.

### Event type comparisons

On day 4, when the greatest number of events were recorded (Table 6 and 7), future group events were strongly predicted by prior group events but not pair events, while future pair events were strongly predicted by prior pair events but not group events. This result suggests that dyadic events and group events are substantially different, and as such, group building processes do not easily emerge from repeated dyadic association but are instead influenced by prior group participation.

## Discussion

The use of automated technology to collect continuous records of animal association data is increasing, and the development of analytical techniques to deal with these large and complex data sets are being explored. We have described a relational event model for animal association data that will allow researchers to analyse continuous event stream data, a form that has become increasingly common in animal studies using autonomous behavioural recording technologies (Wark et al. 2007; Handcock et al. 2009). Understanding and quantifying the social structure of animal populations allow general hypotheses regarding evolutionary and ecological aspects of within- and between-species interactions to be tested (Pinter-Wollman et al. 2014). The current form of the relational event model links temporal variances in event sequence animal association data with changes in social behaviour. This approach allowed us to explore changes in social behaviour associated with the introduction of an unfamiliar individual into an established familiar dyad. By comparing different historical periods, it was possible to determine the temporal patterning and characteristics of a stable relationship and to determine when there were deviations from regular patterns of association.

By modelling distant and recent histories, the relational event model provides insight into how relationships (i.e. stable association patterns) are formed, reproduced or eroded over time. Distinguishing between dyads and groups as predictors of stable association patterns allowed us to show how pairs of familiar steers were more likely to maintain their status quo, as shown by the relatively stable level of dyadic associations in both the long-term (day) and short-term (hourly) relations. This stability reflects a relationship between the familiar pair, where the pattern of encounters in the past is reproduced and future encounters are expected. Preferential social associations exist in many species, for example in cattle (Reinhardt and Reinhardt 1981), pigs (Durrell et al. 2004), giraffes (Carter et al. 2013), dolphins (Lusseau et al. 2003) and finches (Oh and Badyaev 2010). The existence of preferential pair associations in animal systems influences the likelihood of ecologically important processes, such as mate choice and sexual selection, cooperation including social grooming and cooperative foraging, and social learning, which is a major contributor to within-group behavioural adaptation over generations (Coussi-Korbel and Fragaszy 1995). These behaviours have important outcomes for group cohesion and reproductive success.

The dynamic social situation of introducing an unfamiliar into a familiar pair provided the necessary conditions to create a change in social interaction. The model outputs showed within-group variability and volatility in short-term group associations, representative of social disruption caused by the unfamiliar animal’s introduction. The presence of an unfamiliar animal is considered a threat to an individual’s social status as well as competition for resources (Price 2008), resulting in social stress caused by disruption of the existing social hierarchy (Syme and Syme 1979; Zayan 1990). Familiar animals provide each other with social support, which has been shown to provide a calming influence in stressful situations (Boissy and LeNeindre 1997) and protection from outside threats (Neisen et al. 2009). Moreover, the introduced animal is faced with social stressors additional to those experienced by the familiar individuals, which could include an unfamiliar environment, separation from familiar group members, a change in group size and human handling (Zayan and Dantzer 1990; Newberry and Swanson 2001). Thus, it is expected that the introduced individual may seek to form a relationship with conspecifics to alleviate social stress, as being isolated is stressful and individuals are vulnerable without the presence of conspecific to provide social support (Boissy and LeNeindre 1997; Boissy and Dumont 2002). The model outputs provide evidence that the unfamiliar steer was actively trying to engage with either of the familiar animals while there was also evidence to suggest that the familiar pair were excluding the unfamiliar animal; however, as there were no visual observations to validate these claims, our assumptions are speculative but evidenced by the patterns shown in the results. The unfamiliar was the predominant actor creating group events, either joining an existing familiar pair or being approached by the other familiar while already within close proximity to the other familiar steer. Such patterns are suggestive of information seeking behaviour, where individuals change configuration to learn each other’s characteristics and features. Similar occurrences were seen in a study by Psorakis et al. (2012), who used co-location at feeding events to document the change in avian associations as mating pairs were formed at the beginning of a breeding season; association patterns rapidly changed from random to a highly structured proximity network at the onset of the breeding season.

The results demonstrate that inherent differences exist between dyadic and triadic relationships; prior pair-wise interactions were more likely to lead to future pair events and, equally, prior group events lead to future group events. Dyadic interaction forms the basis of a social system (Wasserman and Faust 1994), yet Chase et al. (2003) determined that isolated dyadic outcomes were insufficient to explain group structure and showed that social context influences group outcomes. Triads have unique social properties that are not common to any other group size (Simmel 1950); in human groups, triads present an opportunity for social choice where two can join forces to exclude the third (Feinman and Lewis 1984), while Chase (1982) showed that in chickens, triadic outcomes predominate group behaviour even when four or more individuals are present. The model outcomes suggest that triadic, rather than dyadic, interactions are responsible for group building processes. Future work will use larger group sizes to investigate: if the same pair and group patterns are observed; if triadic interaction forms the basis of group activity; and if the environment (both social and physical) influences association patterns.

The relational event model is well suited to investigations where social connectivity is related to population outcomes that are linked to social factors such as cooperation (Clutton-Brock 2009), social learning (Franz and Nunn 2009; Hoppitt and Laland 2011) or disease transmission (Hamede et al. 2009; Hirsch et al. 2013), as well as physiological events such as oestrus or parturition (Finger et al. 2014). The process of social transmission can be traced through the network, whether it be a series of gradual changes or a specific behavioural event, and individual relationships can be correlated with the rate that it spreads through the population. Further information on individual propensities to interact with the same- or mixed-species individuals is also possible with the relational event model framework; hypotheses on the ecological advantage of inter-species associations could be tested and add value to existing network analyses, such as flock diversity and cooperative foraging (Farine and Milburn 2013) or the process of patch discovery based on network connectivity (Aplin et al. 2012). The familiar-unfamiliar setting is representative of species that exhibit fission-fusion dynamics, where group cohesion varies with time and membership frequently changes (Aureli et al. 2008). Including weighted parameters in the model to predict the likelihood of future events based on prior knowledge creates an informed benchmark with which to compare and contrast association changes. Changes in association patterns leading to the fission event and equally, observations of the process of relationship development as fusion occurs can be assessed. Specific change points can then be related to ecological factors, such as assortativity by sex or degree (fission, e.g. Ramos-Fernández and Morales 2014) or reproduction (fusion, e.g. Psorakis et al. 2012).

The relational event model is very well suited for data structures that are common in the study of animal behaviour. Electronic loggers or recordings of animal interactions provide reliable, highly granular information about social encounters between animals. For example, Swain and Bishop-Hurley (2007) investigated maternal behaviours using proximity loggers to reveal that maternal behaviours extended beyond direct offspring. Methods for the analysis of ordered sequences of interaction data currently require prior transformation. As mentioned previously, aggregation can result in lost information about timing and sequence (Psorakis et al. 2012), which may include data related to biologically important events. The current application of the method does, however, require some a priori definitions to infer meaning from the data. Imposing restrictions on the data collected by autonomous data recording devices is often unavoidable as most devices initially require boundaries to be set that define the activity of interest. For example, proximity loggers require an encounter distance to be set by means of signal strength, thus we deliberately chose a 4 m radius to represent meaningful social contact based on the size of the animal and type of encounters under study. Additionally, 1 s interactions have been shown to over-represent the amount of time two individuals spend within close proximity (Prange et al. 2006) and were therefore discarded. Thus, before any analysis has taken place, we have defined an association to represent proximity within 4 m for longer than 1 s; these settings are based on previous testing and validation to provide the most accurate data representative of the behaviours of interest. Restrictions for other technologies may not necessarily relate to behavioural classifications, for example, the accuracy of global positioning systems (GPS) increases with the frequency at which positional estimates are recorded, known as ‘fix rate’ (Swain et al. 2008). However, issues of battery life and data storage are encountered and thus researchers face a trade-off between data quality and quantity. It is inevitable then that further processing is required to ‘make sense’ of the data. The relational event model, however, is capable of using the data in its most simplistic form with minimal processing enabling researchers to model actual interactions between animals, rather than assuming relationships.

The current relational event model extends the notions of persistence and recency presented in Butts’s (2008) original paper by introducing two time frames of past interactions (short term and long term) for which different statistics are calculated. The relational event model can be applied to any empirical context where a number of social actors interact together over time. Even though in our illustrative example the networks were small (three animals), these were in fact full networks; the relational event model should typically be used when information about the interactions between social actors in a full network is known, including when the composition of actors in the network changes over time. Limitations, however, were imposed on the current model to illustrate its capabilities, and as such, a simpler design of the model has been presented here detailing onsets only. Further refinements and specifications of the current model could include investigating historical periods additional to hour and day, incorporating offsets to investigate social dissolution, and considering the duration of encounter events, to address specific behavioural questions. The parameters presented in the model (pair, group and triangle events) are common structures in animal social networks, and as such, can generally be applied to event data from various animal species. Increasing the social context from triads to larger groups, however, requires additional methodological and biological considerations, for example, additional parameters within the degree distribution, such as three paths and four cycles, as well as full triangles would need to be included. Furthermore, if the interaction between animals has a direction (i.e. that an interaction from A to B is different from an interaction from B to A), then parameters that investigate processes of reciprocity, indegree and outdegree distribution or different forms of closure would also need to be considered. Incorporating goodness of fit criteria into future models could be used to indicate how well the model describes the data. Empirical or simulations studies of biologically important events, such as mating events as evidenced by a sharp increase in male-female interaction or territorial displays as indicated by a sudden increase in male-male encounters, provide appropriate data sets to further test applications of the relational event model.

The results presented in this paper demonstrate how relational event modelling may be used to understand animal social dynamics. We are hopeful that animal behaviour researchers will apply these methods to a broader range of data to investigate animal contact sequences. Proximity loggers and related data collection methods, such as positional locating devices, provide ideal time-stamped association data for relational event modelling. Retaining the sequential nature of social associations provides a fine-grained appreciation as to how social dynamics unfold. We encourage animal behaviour researchers to consider these new event-based statistical models recently introduced into the social networks literature.

## Electronic supplementary material

Below is the link to the electronic supplementary material.

ESM 1 (TXT 13 kb)

ESM 2 (TXT 11 kb)
